# Advances and Challenges in Tissue Engineering: Biomaterials, Cellular Strategies, and Clinical Applications

**DOI:** 10.3390/jfb17040184

**Published:** 2026-04-10

**Authors:** Rosana Farjaminejad, Samira Farjaminejad, Franklin Garcia-Godoy, Anand Marya, Ludovica Nucci, Abdolreza Jamilian

**Affiliations:** 1 Department of Health Services Research and Management, School of Health and Psychological Sciences, City, University of London, London WC1E 7HU, UK; rosana.farjaminejad@city.ac.uk (R.F.); samira.farjaminejad@city.ac.uk (S.F.); 2 Department of Bioscience Research, Bioscience Research Centre, College of Dentistry, University of Tennessee Health Science Centre, 875 Union Avenue, Memphis, TN 38163, USA; fgarciagodoy@gmail.com; 3 Dental School, University of Greater Manchester, Bolton BL3 5AB, UK; amarya@puthisastra.edu.kh; 4 Faculty of Dentistry, University of Puthisastra, Phnom Penh 12211, Cambodia; 5 Department of Mental and Physical Health and Preventive Medicine, University of Campania Luigi Vanvitelli, 80138 Naples, Italy; italy.ludortho@gmail.com

**Keywords:** tissue engineering (TE), skin tissue engineering, bone tissue engineering, cartilage tissue engineering, dental tissue engineering, nerve tissue engineering, liver tissue engineering, cardiac tissue engineering, ophthalmic tissue engineering

## Abstract

Tissue engineering integrates concepts from medicine, biology, and engineering to create living constructs capable of repairing, replacing, or supporting damaged tissues. This multidisciplinary field relies on the interplay between biomaterials, cellular sources, and bioactive signaling to achieve functional tissue regeneration. This review provides a comprehensive overview of recent advances in scaffold design, highlighting natural, synthetic, and hybrid materials, as well as innovative fabrication techniques such as electrospinning, 3D bioprinting, and smart biomaterials. It discusses the role of stem cells and growth factors in directing regeneration and examines a wide range of clinical applications, including skin regeneration, cartilage repair, bone tissue engineering, dental and periodontal regeneration, nerve repair, cardiac tissue engineering, liver tissue models, and ophthalmic applications. Current challenges, such as immune responses, limited vascularization, scalability, and regulatory barriers, are addressed alongside emerging strategies aimed at improving clinical translation. By integrating diverse tissue types and engineering approaches within a unified framework, this review offers a broad yet detailed perspective on the current state and future directions of regenerative medicine.

## 1. Introduction

Tissue engineering (TE) brings together principles from medicine, biology, and engineering to develop living constructs capable of repairing, replacing, or supporting damaged tissues [1]. To function effectively, these engineered materials must do more than simply replicate the shape of native tissues; they need to support cellular attachment and survival, enable oxygen and nutrient delivery, and facilitate waste removal during regeneration [2]. In recent years, growing attention has been directed toward creating advanced platforms that closely mimic the structural and physicochemical characteristics of the native extracellular matrix (ECM) [2]. Achieving this level of biomimicry requires close collaboration across disciplines, with researchers from nanotechnology, biotechnology, and material science contributing to scaffold design and fabrication. Advances in nanotechnology have enabled the development of scaffolds that more precisely replicate ECM architecture at the nanoscale, enhancing cell–material interactions and biological performance [3]. Various fabrication techniques, such as solvent casting [4], freeze drying [5], self-assembly [6], template synthesis [7], phase separation [8], melt blowing [9], and electrospinning [2] have been explored to achieve scaffolds with optimal biofunctionality. As the field continues to evolve, such strategies are bringing us closer to clinically viable solutions for tissue repair and regeneration.

This review aims to summarize the latest advances in TE, focusing on the synergistic roles of biomaterials, scaffolds, cellular sources, and growth factors in promoting tissue regeneration. It explores a wide range of clinical applications, including skin regeneration, cartilage repair, bone TE, dental and periodontal regeneration, nerve repair, cardiac TE, liver tissue models, and ophthalmic applications. The review also addresses current challenges hindering clinical translation and highlights emerging technologies that may shape the future of regenerative medicine. The success of TE approaches, whether in cell-based therapies, artificial organs, or complex tissue replacements, relies on the development of multifunctional biomaterials with finely tuned surface chemistry and mechanical properties. These features must be tailored not only to support cell viability and guide tissue growth in situ, but also to match the mechanical behavior of the target tissue [10,11]. By integrating diverse tissue types and engineering strategies into a unified framework, this work provides a holistic perspective that links fundamental principles of scaffold design, cellular biology, and bioactive signaling with their translation to a wide spectrum of clinical applications. It combines recent experimental findings with advanced fabrication methods such as 3D bioprinting, hybrid scaffolds, and smart biomaterials, while also addressing translational challenges to bridge laboratory innovations with clinical practice. Rather than discussing each tissue independently, this review aims to identify shared design principles and translational barriers that shape the future of scaffold-based regenerative strategies.

## 2. Biomaterials and Scaffold Strategies

The design of scaffolds in TE relies heavily on the selection of appropriate biomaterials, which directly influence biocompatibility, biodegradability, mechanical integrity, and cell–material interactions. Biomaterials are generally categorized into natural, synthetic, and hybrid polymers, each offering specific benefits and limitations that make them suitable for different TE applications [12]. Natural polymers, such as collagen, gelatin, chitosan, alginate, and hyaluronic acid (HA), are derived from biological sources and closely mimic the components of the ECM. Their inherent bioactivity promotes cell adhesion and signaling, while their biodegradability and biocompatibility make them highly attractive for regenerative applications. However, natural polymers often present challenges in terms of mechanical strength, batch-to-batch variability, and thermal stability [13]. In contrast, synthetic polymers like poly (lactic acid) (PLA), poly (glycolic acid) (PGA), poly (lactic-co-glycolic acid) (PLGA), poly (ε-caprolactone) (PCL), and poly (glycerol sebacate) (PGS) offer greater control over mechanical properties, degradation rates, and structural uniformity [14,15,16].

These materials are easier to process and modify chemically, making them suitable for a wide range of applications [16]. However, synthetic scaffolds often lack the biological cues necessary for optimal cell response. To address these limitations, hybrid scaffolds have emerged as a promising strategy, combining the structural advantages of synthetic materials with the bioactivity of natural polymers [17]. Hybrid scaffolds are preferred over purely synthetic ones because they combine the mechanical strength and tunable degradation of synthetic polymers with the bioactivity and cell-friendly properties of natural materials. This synergy improves biocompatibility, supports better cell adhesion and proliferation, and enhances tissue regeneration, especially in complex environments like bone, where both structural support and biological signaling are essential [18].

Recent innovations have driven the development of smart biomaterial-engineered systems capable of responding to environmental cues such as pH, temperature, or mechanical stimuli [19]. These biomaterials can be functionalized with bioactive molecules (e.g., growth factors, peptides) and designed to support dynamic interactions with cells during tissue regeneration [20]. Notably, advancements in 3D bioprinting have revolutionized scaffold fabrication, enabling the precise deposition of cells and biomaterials (bioinks) in predefined architectures [21]. Bioprinting techniques, such as extrusion-based printing, stereolithography (SLA), and selective laser sintering (SLS), enable the construction of patient-specific implants with tunable porosity, shape, and biochemical composition. These techniques enhance the capacity to mimic native tissue microenvironments and are increasingly used in the design of complex structures for bone, cartilage, and organ regeneration [22,23].

One of the major challenges in scaffold design is achieving proper vascularization and mechanical compatibility with host tissues. Effective vascular integration is essential for nutrient and oxygen supply, especially in large or dense tissue constructs [24]. Scaffold features such as interconnected porosity, appropriate pore size, and surface topography significantly influence cellular infiltration and angiogenesis [25]. From a mechanical standpoint, scaffolds must match the stiffness and elasticity of the target tissue to provide appropriate structural support and mechanical signaling [26]. For example, bone scaffolds require higher compressive strength, while soft tissues like cardiac or neural constructs benefit from more flexible materials [27]. Advances in scaffold fabrication methods, such as electrospinning, gas foaming, and phase separation, allow for fine-tuning of these properties, enabling the creation of scaffolds that are both mechanically stable and biologically instructive [28].

## 3. Cellular Sources and Growth Factors

TE is fundamentally based on two biological foundations: choosing appropriate cellular sources and incorporating essential growth factors that regulate regeneration and direct tissue-specific results [29]. Among cellular sources, mesenchymal stem cells (MSCs) are the most versatile and widely used due to their ability to differentiate into multiple lineages, their immunomodulatory properties, and their relative ease of extraction from bone marrow, adipose tissue, or the umbilical cord [30]. These cells have been extensively used for bone regeneration, cartilage repair, nerve healing, and dental reconstruction [31,32,33]. Adipose-derived stem cells (ADSCs), which can be obtained with minimal invasiveness, show potential for soft tissue repair and skin applications [34]. Induced pluripotent stem cells (iPSCs) are increasingly recognized for their ability to create more complex tissue models, such as the liver [35], cardiovascular [36], and ophthalmic tissues [37], due to their pluripotency and capacity to produce patient-specific cell types. Growth factors act as biological mediators that play pivotal roles in regulating cellular behavior, promoting angiogenesis, and supporting the development of tissue-specific architecture. Integrating stem cell technologies with precise growth factor signaling is crucial for developing functional tissue replacements [38]. Ongoing research into delivery systems, such as bioactive scaffolds and controlled-release carriers, aims to refine the spatial and temporal dynamics of these biological signals, ultimately improving clinical outcomes across various TE applications [39].

## 4. Applications for Tissue Engineering

TE has emerged in recent years as a multidisciplinary research field with applications in skin, bone, cartilage, dentistry, nerve, cardiac, liver, and ocular regeneration. By creating optimized microenvironments, this approach accelerates tissue repair and regeneration. Although each tissue type requires its specific strategy, a common principle across all applications is achieving a balance between biocompatibility, mechanical performance, and biological signaling to ensure functional integration and long-term stability in vivo. The details of these applications are fully described in the following sections and summarized in Table 1.

### 4.1. Skin Tissue Engineering

Skin TE has become a vital solution for treating extensive wounds, burns, and chronic skin injuries, particularly in situations where traditional therapies such as autografts, despite being the clinical gold standard, are constrained by donor site morbidity or limited graft availability. The primary goal is to restore the skin’s multilayered structure and function through coordinated matrix remodeling and regenerative strategies [91]. A key element in the development of tissue-engineered skin substitutes is the use of scaffold matrices engineered to support three-dimensional cell growth. Designed to be biocompatible, biodegradable, and suitable as wound dressings, these scaffolds provide an optimal environment for tissue repair. Recent advances have shifted towards seeding scaffolds with specific cell populations, such as keratinocytes and fibroblasts, to further promote and refine regenerative outcomes [40]. In the context of skin regeneration, growth factors such as epidermal growth factor (EGF) and vascular endothelial growth factor (VEGF) play a pivotal role in promoting re-epithelialization and stimulating neovascularization within wound beds [92,93,94].

#### Scaffolds in Skin Tissue Engineering

Scaffolds are essential to skin TE, functioning as three-dimensional matrices that support cell adhesion, migration, proliferation, and differentiation while directing new tissue formation [95]. They are fabricated from a wide range of natural and synthetic biomaterials, used individually or in combination to balance biocompatibility, biodegradability, and mechanical strength. Natural biomaterials, such as collagen, cellulose, and chitosan, are protein- or polysaccharide-based and closely mimic the native ECM, making them highly biocompatible, readily degradable, and well-suited for skin cell growth. In contrast, synthetic biomaterials, including nanomaterials like polyvinylpyrrolidone (PVP), PCL, polyethylene glycol (PEG), and PLA, provide mechanical strength and structural stability [40]. Combining these materials in composite scaffolds, such as PCL–collagen matrices or silk fibroin–polyurethane (PU) blends, merges biological cues with enhanced durability. Collagen remains the most widely used natural scaffold due to its ECM-like architecture, while clinically applied acellular dermal matrices (e.g., Integra^®^, AlloDerm^®^) also leverage preserved ECM structure to support host cell infiltration and gradual tissue remodeling [40].

Notably, blood plasma, fibrinogen, and fibrin have also been highlighted as promising natural biomaterials for skin tissue engineering, demonstrating strong preclinical wound-healing potential, although their clinical translation remains limited [96]. Examples of commonly used natural and synthetic scaffolds for skin TE are summarized in Table 2.

In skin TE, several fabrication strategies have been developed, with four primary approaches: ECM-secreting cell sheets, pre-fabricated porous scaffolds made from synthetic, natural, or biodegradable materials, decellularized ECM scaffolds, and cell-laden hydrogels that provide a hydrated, supportive 3D environment [40]. Electrospun gelatin nanofiber scaffolds have shown promising potential for wound healing applications [97]. Various gelatin-based formulations for wound and burn dressings, such as gelatin–alginate sponges [98], gelatin incorporated with EGF [99], and gelatin films, have also demonstrated efficacy in the treatment of skin injuries. To further improve scaffold performance, researchers have explored the incorporation of nanoparticles, which can enhance antibacterial activity, angiogenesis, and cell proliferation (Table 3). For example, poly(3-hydroxyoctanoate)-fabricated nano-sized 45S5 Bioglass^®^ scaffolds have been reported for use in wound dressings, as they provide a favorable environment for tissue growth, offering properties such as wettability and rough surfaces, and can accelerate blood clot formation [100]. Similarly, Chua et al. reported that composite scaffolds that integrate natural polymers such as collagen with synthetic components like elastin or glycosaminoglycans have shown enhanced vascularization, mechanical stability, and long-term integration in clinical burn treatments, underscoring the potential of combining bioactivity with tunable degradation profiles [101].

**Table 2:** Overview of natural and synthetic scaffolds used in skin tissue engineering and their functional roles in wound repair.

Biomaterial	Material Type	Representative Examples	Role in Skin Regeneration	References
Collagen	Natural polymer	Orcel^®^ (bilayered bovine collagen sponge with keratinocytes & fibroblasts), Terudermis^®^ (bovine collagen + silicone layer), Pelnac^®^ (porcine collagen sponge)	Serves as dermal or dermo-epidermal substitute; supports keratinocyte/fibroblast growth; promotes growth factor production, angiogenesis, and wound healing; used for burns, trauma repair, tumor excision defects, and necrotizing fasciitis	[102,103]
Gelatin	Natural polymer	Gelatin–alginate sponges, gelatin + EGF dressings, gelatin films, photo- crosslinkable gelatin hydrogel with keratinocytes, gelatin-based microspheres	Less immunogenic than collagen; promotes enhanced cell adhesion via RGD sequences; used in wound and burn dressings, epidermal substitutes, and as microcarriers for stem cells; supports angiogenesis, cell proliferation, and sweat gland repair	[40,104]
Chitosan	Natural polymer	Chitosan nanofibrous scaffolds; Terudermis^®^, Pelnac^®^ (with collagen), Biobrane^®^, Integra^®^, Apligraf, Transcyte	Enhances adhesion, growth, and differentiation of keratinocytes, fibroblasts, and endothelial cells; supports wound healing and tissue repair; often combined with collagen or other polymers in commercial scaffolds	[102,105,106]
HA	Natural polymer	HA-based hydrogels (Hyaff^®^, Laserskin^®^, Hyalograft^®^); HA nanofibers via air-blowing	Key ECM component in connective tissue; promotes scar-free wound healing, enhances epithelial cell proliferation, regulates macrophage activity, supports angiogenesis and neurite repair; the nanofiber form improves electrospinning limitations and accelerates healing	[97,106,107,108]
Silk fibroin	Natural polymer	Silk fibroin, electrospun mats, SF–PU composites	Mechanical strength, biocompatibility	[109]
Alginate	Natural polymer	Alginate-based hydrogel dressings	Hydrogel scaffold, wound dressings	[110]
PCL	Synthetic polymer	Electrospun PCL nanofibers; PCL blended with PLA or PGA	Widely used in wound dressings and skin regeneration scaffolds due to biodegradability, mechanical strength, and ability to be fabricated into porous structures; often combined with other polymers for improved cell interaction and vascularization	[40,111,112]
PLGA	Synthetic copolymer	PLGA microspheres loaded with growth factor and gentamicin	Enable controlled drug/protein release, promote fibroblast adhesion and proliferation, and antibacterial activity, and support MSC-mediated wound healing and sweat gland repair	[113]
PU	Synthetic polymer	Composite PU foams with hydroxyapatite and silver sulfadiazine; fatty acid-based PU films; biodegradable PU blends (e.g., poly-3-hydroxybutyrate–PCL)	Strong mechanical support, high absorbency, antibacterial activity, and effective wound dressings that reduce wound size in vivo	[111,114,115]
Fibrin	Natural polymer	ADSC–fibrin scaffolds	Promotes angiogenesis and healing	[40]
Keratin	Natural protein from animal sources	Keratin dressings with antibiotics	Incorporated into dressing materials for controlled release of antibiotics or growth factors, promoting wound healing	[116,117]
Bioactive glass	Bioceramic (bioactive glass)	45S5 Bioglass^®^ in PLGA-mesh, nano-45S5 Bioglass^®^ in poly(3-hydroxyoctanoate), MBG fibers with poly (ethylene oxide), chitosan–MBG films	Promotes angiogenesis and neovascularization; supports tissue growth via surface wettability and roughness; accelerates blood clotting; can deliver anti-inflammatory drugs; used in wound dressings and skin repair	[105,118]

Different wound types require different scaffold formats. Porous scaffolds, such as sponges and foams made from collagen, PCL, or PLGA, offer high surface area and interconnected pores that facilitate cell penetration and nutrient diffusion. These are typically fabricated using salt leaching or 3D printing to control porosity and architecture [40]. Fibrous scaffolds, particularly electrospun mats, closely mimic the fibrous structure of natural ECM and have been created using polymers such as chitosan, silk fibroin, and gelatin to support the growth of keratinocytes and fibroblasts [109]. Hydrogel-based scaffolds provide high water content and favorable mechanical properties, making them especially suitable for irregular wound geometries and full-thickness defects. Materials such as alginate, HA, and PEG have been used to create hydrogels capable of controlled drug and growth factor release [110]. These systems can function as injectable materials or wound dressings that conform to wound beds while delivering bioactive cues.

In addition to the scaffold architecture, cellular components play a critical role in tissue restoration. Primary human keratinocytes and fibroblasts are frequently employed to reconstruct the epidermis and dermis, respectively. More recently, MSCs and ADSCs have gained attention for their secretion of pro-regenerative cytokines and promotion of neovascularization. For example, ADSCs embedded in a fibrin scaffold enhanced wound closure and dermal regeneration in diabetic rats [40], while human umbilical cord-derived MSCs in a decellularized dermal matrix improved epithelialization and reduced scar formation [41].

Progress toward clinical translation includes bilayered scaffolds that mimic the epidermal–dermal interface, with keratinocytes on the surface and fibroblasts embedded in a collagen matrix, as well as growth factor integrated systems (e.g., microspheres or surface-immobilized ligands) that enhance vascularization and skin barrier restoration [101,119]. Despite these advances, challenges remain, including poor integration with host tissue, limited sensory recovery, and inadequate vascularization in chronic or extensive wounds. Current research is addressing these limitations through pre-vascularized scaffolds, immunomodulatory hydrogel systems, and patient-specific cell sources such as iPSCs [101,119]. Huang et al. demonstrated that bFGF-loaded collagen scaffolds seeded with bFGF-overexpressing human umbilical cord mesenchymal stromal cells significantly accelerated diabetic full-thickness wound healing through enhanced angiogenesis, collagen deposition, and re-epithelialization, primarily via activation of the HIF-1 signaling pathway [120]. Similarly, bFGF-loaded silk fibroin scaffolds enhanced dermal regeneration and neovascularization in full-thickness defects [42], and TGF-β1-modified hydrogel systems reduced inflammation while improving ECM deposition and collagen synthesis in vivo [43].

These advances highlight the progress of scaffold-based approaches; however, the next frontier lies in smart and dynamic platforms such as 4D bioprinting and Artificial intelligence (AI)-assisted scaffold design, which enable responsive, patient-specific constructs to overcome current challenges in vascularization, integration, and controlled therapeutic delivery [121].

**Table 3:** Nanomaterial-based scaffolds for skin TE.

Therapeutic Application	Material/Composition	Application Description	Study Stage	Key Limitation	Clinical Translation Status	Ref.
General Wound Healing	GO in ASC-derived ECM crosslinked with genipin	Applied as a scaffold for skin tissue regeneration	In vivo (rat model, 4 weeks)	Short-term evaluation; no large-animal or clinical data	Preclinical	[122]
	Gold nanoparticles loaded into PEG and cationic PAH	Scaffold for wound repair animal model	In vivo (rat model, 14 days)	Short-term animal study. No human data	Preclinical study; no clinical validation	[123]
	Nano-silver hydrogel coating film	Scaffold for wound healing in a deep partial-thickness scald rabbit model	In vivo (rabbit model, 18 days)	Animal study only. No human clinical validation	Preclinical	[124]
	GG-g-PAGA hydrogel with AgNPs	Self-healing injectable antibacterial wound dressing hydrogel	In vitro (antibacterial and cytocompatibility assays)	No in vivo or clinical validation. Long-term safety not assessed	Preclinical (laboratory stage)	[125]
	PVA/PEG/chitosan hydrogels containing AgNPs	Antibacterial hydrogel demonstrating enhanced bacteriostatic activity and improved fibroblast viability in vitro	In vitro (cell culture and material characterization)	No in vivo wound model; limited to laboratory evaluation	Preclinical (in vitro only)	[126]
Diabetic Wound Healing	PU/Pluronic F127 nanofibers with peppermint extract and gelatin nanoparticles	Electrospun nanofibrous wound dressing enabling controlled release of peppermint extract with antibacterial and anti-inflammatory effects to enhance diabetic wound healing	In vivo (Streptozotocin-induced diabetic rat model, 21 days)	Limited to small animal models; no human clinical data; long-term safety not evaluated	Preclinical	[127]
	Gelatin/poly-3-hydroxybutyrate nano/microfibers	ECM-mimicking scaffold promoting fibroblast proliferation, accelerated wound closure, and improved skin regeneration in diabetic wounds	In vivo (diabetic rat model, 21 days) + in vitro fibroblast assays	Small animal model only; no human or large-animal validation	Preclinical	[128]
Skin Cancer /Tumor Treatment	Injectable chitosan thermosensitive hydrogel incorporating defective black TiO_2_–x nanoparticles (~50 nm)	Bifunctional injectable thermogel enabling combined photothermal and photodynamic therapy for tumor ablation while simultaneously promoting skin regeneration	In vitro + in vivo (murine skin tumor and chronic wound models)	Murine model only; laser-dependent therapy; no long-term safety or human data	Preclinical	[129]
	Molybdenum oxide (MoO_3_) nanoparticles incorporated into electrospun polycaprolactone (PCL) nanofibers	Selectively induces apoptosis in skin cancer cells, reduces >50% cancer cell viability, and suppresses tumor progression (~30%) in vivo	In vitro (A431, HT1080, G361 cell lines) and in vivo (zebrafish tumor model, 14 days)	Preliminary non-mammalian model; no large-animal or clinical validation	Preclinical	[130]
Skin Regeneration and Stimulation	PCL/PU composite with graphene oxide	Supports human skin fibroblast adhesion, proliferation, and improves scaffold hydrophilicity and biocompatibility	In vitro (MTT assay, contact angle, SEM characterization)	No in vivo validation; limited to short-term cell studies	Preclinical (in vitro only)	[131]
	Collagen type I hydrogel incorporating silica core–shell nanoparticles dual-loaded with gentamicin and rifamycin	Sustained dual antibiotic release system for prevention of wound infection and reduction in bacterial load in cutaneous wounds	In vivo (rat superficial skin infection model, 48 h)	Short-term evaluation; no large-animal or clinical studies	Preclinical	[132]
	Smart polymeric nano-drug (SPN) containing cerium dioxide nanoparticles; smart polymeric nano-drug combined with mesenchymal stem cells (SPN + SC)	Topical polymer-based nano-drug applied to full-thickness skin wounds in aged Wistar rats to enhance wound closure, reduce inflammation, accelerate transition from exudation to proliferation phase, and promote epithelialization and tissue regeneration	In vivo (aged Wistar rat full-thickness wound model)	Animal model only; no human clinical validation; short-term follow-up	Preclinical	[133]
	Keratin–chitosan/n-ZnO nanocomposite hydrogel	Antibacterial burn wound dressing with enhanced porosity, mechanical strength, fibroblast viability, and accelerated wound closure	In vitro (fibroblast cells) and in vivo (SD rat burn model, 14 days)	Small animal model only; no large-animal or clinical data	Preclinical	[134]
	Injectable glycol chitosan/silica nanoparticle (GC/SiNP) composite hydrogel	Adhesive, injectable wound dressing supporting fibroblast encapsulation, enhanced vascularization, hair follicle regeneration, and reduced scar formation in full-thickness skin defects	In vivo (mouse full-thickness wound model)	Small animal model only; no long-term safety or human validation	Preclinical	[135]

### 4.2. Bone Tissue Engineering

Bone tissue engineering (BTE) relies on a triad of components: progenitor cells, bioactive signaling molecules, and scaffolds that provide essential structural and biological cues for new bone formation [136]. Among these, skeletal stem cells (SSCs) have emerged as pivotal elements due to their intrinsic ability to differentiate into multiple lineages, including osteoblasts, chondrocytes, and adipocytes [137]. The concept of SSCs was first confirmed by Friedenstein and colleagues, who demonstrated that colony-forming unit-fibroblasts (CFU-Fs) from bone marrow stromal cells could generate bone in vivo. Bianco et al. proposed that SSCs reside in the perivascular niches of bone marrow and can form complete heterotopic ossicles, including bone and hematopoietic marrow, when transplanted [2,138].

#### 4.2.1. Bioactive Molecules and Growth Factor Delivery

Different factors that promote tissue growth have been found at the skeletal damage site and have a physiologic role in healing bone fractures. Osteoinductive GFs such as platelet-derived growth factors (PDGFs), bone morphogenic proteins (BMPs), insulin-like growth factors (IGFs), transforming growth factors (TGFs-ß), and VEGFs have presented great application potentials in bone healing and osteogenesis for regulating cell behavior, including recruitment, migration, adhesion, proliferation, and differentiation [139]. The incorporation of growth factors into scaffolds further amplifies their regenerative potential. BMP-2 is one of the most widely studied osteoinductive agents and has been shown to significantly enhance stem cell recruitment, proliferation, and osteogenic differentiation when loaded into PLGA or gelatin-based scaffolds [140]. For example, gelatin-based hydrogels and poly PLGA scaffolds loaded with BMP-2 have shown increased bone mineralization and improved defect healing in various preclinical models [141]. Chai et al. developed an injectable scaffold system using photo-crosslinked GelMA hydrogel loaded with bone marrow mesenchymal stem cells (BMSCs) and bone morphogenetic protein 2 (BMP2) and demonstrated that this combination significantly enhanced osteogenic differentiation and bone defect repair in vivo [142]. Similarly, VEGF has been integrated into scaffold systems to stimulate angiogenesis, which is essential for the survival of large tissue-engineered constructs. Studies have shown that co-delivery of VEGF and BMP-2 enhances both vascularization and osteogenesis, leading to more robust and stable bone formation [143]. These combined approaches emphasize the importance of delivering both osteogenic and angiogenic signals to recapitulate the complexity of bone healing.

#### 4.2.2. Scaffold Materials in Bone Tissue Engineering

Scaffolds used in BTE must replicate the natural ECM in terms of both structure and function. These scaffolds provide a three-dimensional environment that facilitates cell adhesion, proliferation, and differentiation [144]. A wide range of natural and synthetic polymers has been explored for this purpose. Natural polymers such as collagen, chitosan, and alginate offer excellent biocompatibility and bioactivity, but are often limited by poor mechanical strength and rapid degradation [145]. Several studies have demonstrated the efficacy of specific scaffold systems in promoting bone regeneration. Farjaminejad et al. developed novel bio-rubber scaffolds based on poly (glycerol sebacic acid) modified with succinic acid and ε-caprolactone, reinforced with nano-hydroxyapatite and nanoclay, enabling precise tuning of hydrophilicity, mechanical strength, and degradation rate to optimize performance for bone regeneration applications [15].

Wang et al. fabricated a 3D-printed PLA scaffold incorporating nano-hydroxyapatite (nHA), which significantly enhanced mineralization and vascular infiltration when tested in vivo [146]. Similarly, Diao et al. developed β-tricalcium phosphate (β-TCP) scaffolds with gradient pore sizes (ranging from 300 to 500 µm) and observed enhanced migration and differentiation of MSCs, supporting better integration with host tissue [147]. Lee et al. also discussed the impact of scaffold architecture, highlighting how mechanical stiffness, pore size, and rough surface influence osteogenic differentiation [46].

**Figure 1:**
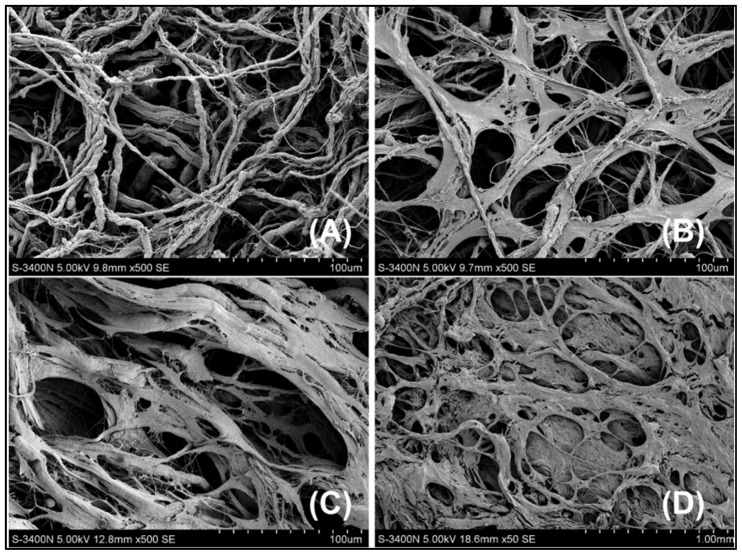
Representative SEM images showing the sequential attachment and growth of human bone marrow-derived MSCs on type I collagen fibers derived from human skin. (**A**) Before cell seeding, (**B**) 8 h after seeding, cells adhere to collagen fibers; (**C**) 24 h after seeding, ECM formation and cell ingrowth into the collagen fiber network are observed; (**D**) 48 h after seeding, complete integration of cells, ECM, and the collagen scaffold is achieved. Reprinted from Ref. [44].

Collagen type I, particularly when functionalized with the P-15 cell-binding domain, provides a biomimetic and highly effective environment for promoting the adhesion, proliferation, and differentiation of MSCs [148]. These fibrous scaffolds not only serve as a suitable substrate for cell growth but also actively participate in osteogenesis by guiding the formation and spatial arrangement of carbonated apatite minerals. Moreover, under cellular stress conditions, the collagen matrix can stimulate MSC migration to injury sites through the release of chemotactic signals [149,150]. As shown in Figure 1, scanning electron microscopy (SEM) images illustrate the sequential stages of cell–scaffold interaction: (A) before cell seeding, the fibrous structure of collagen is clearly visible; (B) 8 h after seeding, cells adhere effectively to the collagen fibers; (C) at 24 h, ECM formation and cell ingrowth into the collagen fiber network are evident; and (D) after 48 h, complete cell–matrix–scaffold integration is achieved.

Nanoparticles in bone TE have introduced new dimensions to scaffold development by improving structural integrity, biological signaling, and drug delivery capabilities [151]. In a recent review, Farjaminejad et al. outlined the role of nano-hydroxyapatite in mimicking the mineral phase of bone and enhancing osteoconductivity [47].

Recent advances suggest that integrating electrical stimulation (ES) into BTE can enhance osteogenesis and angiogenesis via BMP and Wnt/β-catenin pathway activation. Piezoelectric scaffolds, such as PVDF/PVDF-TrFE, BaTiO_3_-reinforced polymers, and β-PCL nanofibers, generate endogenous electrical cues under mechanical loading, mimicking bone’s bioelectric environment and promoting osteoblast activity [152].

Despite substantial advancements, several challenges remain in translating these strategies into clinical applications. Variability in stem cell populations, immune responses to synthetic materials, inconsistent scaffold degradation rates, and the complexity of vascular integration continue to limit the routine use of BTE in clinical settings. Black et al. emphasized the importance of aligning scaffold design with biological requirements and preclinical validation to facilitate regulatory approval and clinical translation [153]. The need for standardization in scaffold fabrication, material testing, and cell characterization remains urgent to bridge the gap between laboratory research and patient treatment [153]. One promising approach to overcoming these challenges is the integration of AI, which can accelerate material discovery, optimize scaffold properties, and reduce experimental time and cost. For example, Mackay et al. (2021) applied a conditional generative adversarial network (GAN) to predict the behavior of Stro-1 selected human bone marrow stromal cells (HBMSCs), successfully reducing culture time by 24 h, demonstrating how AI can directly address resource-intensive steps in bone tissue engineering [154].

Overall, BTE integrates biological and material sciences to develop scaffolds that can restore bone integrity. Specific examples, such as BMP-2-loaded PLGA scaffolds, nano-HA-reinforced polymer matrices, and GO-enhanced constructs, reflect the sophistication of current approaches. With continuous refinement of scaffold composition, cellular selection, and biofunctionalization, BTE holds strong promise for becoming a routine part of regenerative orthopedic and dental therapies.

### 4.3. Cartilage Tissue Engineering

Cartilage TE has emerged as an effective approach for repairing damaged cartilage, particularly articular cartilage, which regenerates poorly due to its limited blood supply and nerve connections, low cell density, and slow matrix regeneration. Conventional clinical methods such as autologous chondrocyte implantation, microfracture, and osteochondral grafts often result in the development of mechanically weaker fibrocartilage and are associated with inconsistent long-term results, donor site complications, and chondrocyte dedifferentiation [51]. Cartilage TE strategies rely on the coordinated use of three essential components: chondrogenic progenitor cells such as bone marrow-derived mesenchymal stem cells (BMSCs), signaling molecules like TGF-β1 to induce chondrogenic differentiation, and biocompatible scaffolds, particularly injectable hydrogels such as GelMA, which provide structural support and promote cell viability, ECM production, and integration with host tissue [52].

MSCs, especially BMSCs, have been extensively studied as a promising cell source for cartilage regeneration due to their strong chondrogenic potential, immunomodulatory functions, and ease of harvesting from bone marrow aspirates. When stimulated with factors like TGF-β1, BMSCs can differentiate into chondrocytes and produce key cartilage ECM components such as collagen type II and aggrecan. Their ability to modulate inflammatory responses and enhance matrix synthesis supports their therapeutic potential in osteochondral repair strategies. Studies indicate that MSCs placed within scaffolds can significantly promote subchondral bone regeneration and improve cartilage integration in defect models [53]. The incorporation of chondrogenic growth factors significantly enhances the functional performance of engineered cartilage. Commonly used factors, such as TGF-β, BMPs, and IGF-1, effectively promote chondrogenic differentiation. Research by Zhao et al. demonstrated that scaffolds infused with TGF-β1 led to increased cartilage matrix deposition and reduced inflammation, while the addition of BMP-7 improved cartilage-specific gene expression and matrix development [52,155].

#### Scaffold Material Cartilage Tissue Engineering

Biocompatible scaffold materials play a crucial role in the survival, growth, and chondrogenic differentiation of MSCs. These scaffolds must provide optimal mechanical strength, feature interconnected porosity, and be biodegradable while mimicking the intrinsic ECM of cartilage [52]. Zhao et al. reviewed various scaffold types used in cartilage TE, including natural polymers like collagen and chitosan, synthetic polymers such as PLGA, and decellularized cartilage matrix. For instance, chitosan-collagen composite scaffolds promoted chondrocyte proliferation and improved histological structure in animal studies [52]. Furthermore, alginate and HA-based hydrogels have been shown to enhance nutrient diffusion and boost matrix production, contributing to improved neocartilage formation [156]. 3D bioprinting is an innovative technique in cartilage TE, allowing for the precise layer-by-layer placement of cells and biomaterials, thus resembling the natural cartilage structure more closely than conventional methods. Among the materials used, GelMA-based bioinks stand out for their excellent cell compatibility and adjustable mechanical properties [157]. Zheng et al. developed GelMA/silk fibroin interpenetrating network hydrogels with enhanced mechanical strength and biocompatibility, which supported BMSC chondrogenesis and promoted in vivo cartilage regeneration, highlighting their potential for functional cartilage repair [158]. Together, these findings highlight the synergistic interaction of MSCs, bioactive scaffolds, and chondrogenic signals in regenerating functional cartilage tissue. Ongoing advancements in biomaterial design and bioprinting technologies are anticipated to further enhance the clinical application of cartilage TE for treating osteoarthritis and cartilage injuries.

### 4.4. Dental and Periodontal Applications

TE has become a groundbreaking technique in modern dentistry, offering innovative strategies for regenerating dental pulp, periodontal structures, and alveolar bone [159]. Conventional treatments like root canal therapy, periodontal surgery, and bone grafting often fall short in restoring the biological and functional integrity of dental tissues [160]. In contrast, TE employs a triad of stem cells, signaling molecules, and scaffolds to overcome these limitations and enhance regeneration [161].

In dental pulp regeneration, scaffolding materials such as collagen, chitosan, HA, and fibrin are utilized to mimic the native ECM and support cellular organization and vascularization [162]. For instance, chitosan/gelatin scaffolds crosslinked with 0.1% glutaraldehyde and seeded with BMP-2 pretreated dental pulp stem cells have been shown to enhance osteo/odontogenic gene expression and promote in vivo mineralized bone formation, supporting their potential for alveolar/orofacial reconstruction [163]. Keswani et al. demonstrated that the clinical application of platelet-rich fibrin in immature permanent teeth led to continued root development, including increased root length and dentinal wall thickness, due to sustained growth factor release that enhances angiogenesis and tissue repair [164]. For periodontal regeneration, stem cells from sources such as the periodontal ligament, dental follicle, and gingiva can be combined with biomaterial scaffolds, such as collagen, chitosan, PLGA, and hydrogels, often supplemented with bioactive molecules like BMP-2 and PDGF to enhance osteogenesis, angiogenesis, and regeneration of the periodontal complex [165]. Mafalda et al. developed electrospun polycaprolactone/chitosan scaffolds loaded with lyophilized extracellular matrix from periodontal ligament stem cells, which maintained favorable physical properties, enhanced PDLSC proliferation, and significantly promoted osteogenic differentiation and mineralization, showing promise for alveolar bone regeneration in periodontal tissue engineering [166]. Injectable hydrogels, particularly GelMA, have gained attention for their ability to deliver stem cells and bioactive factors minimally invasively, supporting extracellular matrix production and integration with surrounding pulp tissue [167].

**Figure 2:**
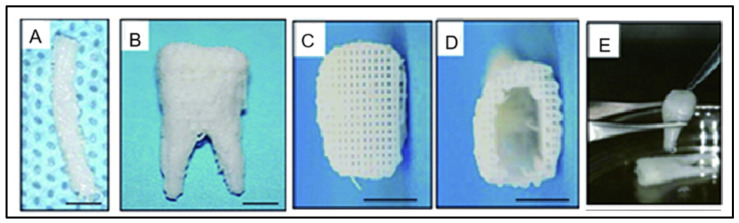
Three-dimensionally bioprinted hybrid polycaprolactone–hydroxyapatite scaffolds replicating a rat mandibular incisor (**A**) and the human mandibular first molar (**B**), featuring 200 µm microstrands with interconnecting microchannels. (**C**,**D**) Human molar scaffold channels (**E**) were infused with stromal-derived factor-1 and BMP-7 in type I collagen before gelation. Reprinted from Ref. [168].

Advanced fabrication methods like 3D bioprinting and electrospinning have enabled the design of anatomically and functionally relevant scaffolds with aligned fibers and gradient structures. These techniques provide spatial control over cell placement and enhance mechanical strength, promoting the regeneration of both soft and hard periodontal tissues [165,168]. An example of such an approach is shown in Figure 2, depicting a 3D-bioprinted hybrid polycaprolactone–hydroxyapatite scaffold with anatomically defined architecture and microchannels to facilitate cell homing and angiogenesis, illustrating the potential of advanced scaffold fabrication for periodontal TE [168]. Cell homing strategies utilizing bioactive molecules, such as stromal cell-derived factor-1 (SDF-1) and VEGF, have gained attention as promising approaches in periodontal and pulp regeneration. When incorporated into scaffolds, these factors can recruit endogenous progenitor cells to defect sites, thereby reducing or eliminating the need for exogenous stem cell transplantation [169]. Nanotechnology has significantly advanced restorative dentistry through the development of nanoparticle-enhanced dental composites. Incorporating materials such as silica, nano-hydroxyapatite, and zirconia improves mechanical strength, aesthetics, durability, and antibacterial properties, while reducing polymerization shrinkage [170]. Clinical evidence supports their safety, biocompatibility, and potential for longer-lasting restorations, although further research is needed to address technical and economic challenges and to develop smart, multifunctional materials for personalized and regenerative care [171].

### 4.5. Nerve Regeneration

**Figure 3:**
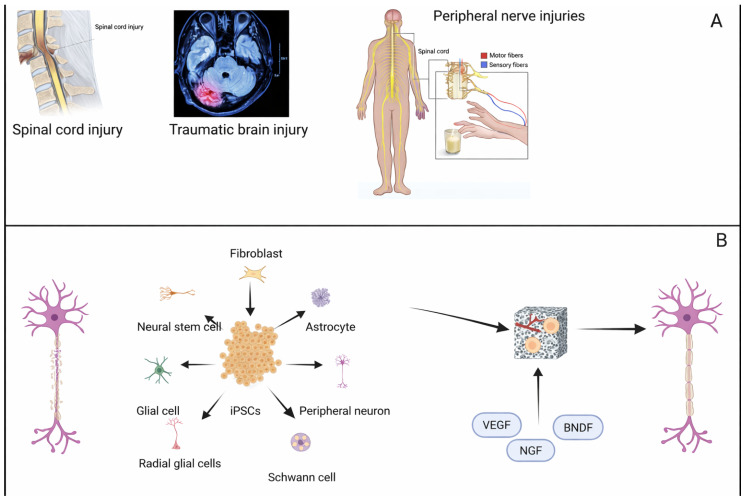
(**A**) NTE aims to restore function in conditions such as SCI, TBI, degenerative diseases, and peripheral nerve injuries, where traditional treatments fall short. By combining biomaterials, cells (e.g., iPSCs, Schwann cells, MSCs), and bioactive molecules (e.g., NGF, BDNF, GDNF, FGFs, VEGF), NTE creates a supportive microenvironment for nerve repair and regeneration. (**B**) Schematic representation of the NTE approach, showing cell sources, biomaterial scaffolds, and neurotrophic factors working together to form an engineered neural construct that promotes neuronal regeneration (Created with BioRender.com).

Nerve tissue engineering (NTE) has emerged as a promising strategy to overcome the limited regenerative capacity of the central and peripheral nervous systems, addressing conditions such as spinal cord injury (SCI), traumatic brain injury (TBI), degenerative diseases, and peripheral nerve injuries (Figure 3A), where conventional treatments often fall short in restoring function [172]. TE combines biomaterials, cells (e.g., iPSC, iPSC-derived neural crest stem cells, Schwann cells, and MSC), and bioactive molecules such as nerve growth factor (NGF), brain-derived neurotrophic factor (BDNF), glial cell line-derived neurotrophic factor (GDNF), FGFs, and VEGF to create a microenvironment conducive to nerve repair and regeneration (Figure 3B) [173].

A central focus in NTE is the design of scaffolds that can mimic the native ECM, support cellular growth, and guide axonal regrowth. Natural polymers such as collagen, gelatin, and chitosan are frequently used due to their biocompatibility and ability to support Schwann cell adhesion and proliferation. For instance, chitosan-based conduits have demonstrated efficacy in promoting neurite extension and enhancing myelination [57]. Synthetic polymers like PLGA and PCL are also widely utilized for their tunable mechanical properties and degradation rates, often modified with nanoparticles or growth factors to enhance bioactivity [58].

Advanced fabrication techniques such as electrospinning and 3D bioprinting have enabled the creation of aligned nanofiber scaffolds that guide axon orientation. Incorporating electrical stimulation and conductive materials, including graphene and polypyrrole (PPy), further promotes neurite outgrowth and functional recovery. One study reported the integration of Schwann cells into electrospun PLGA scaffolds, resulting in improved axonal regrowth in a rat sciatic nerve model [58]. Moreover, recent work has emphasized the role of immune modulation in successful nerve repair. Creating scaffolds that modulate the local immune response, such as those releasing anti-inflammatory agents or promoting M2 macrophage polarization, has been shown to significantly enhance regeneration outcomes. This immunomodulatory approach, combined with scaffold topography and biochemical signaling, provides a holistic strategy for addressing both peripheral nerve injuries and central nervous system trauma [174].

Feng et al. developed a chitosan conduit filled with regulatory T cells (Tregs) and supported by a nano artificial growth factor hydrogel containing a cyclic BDNF mimetic sequence for a 6 mm sciatic nerve defect in mice. This approach preserved Treg stability and Foxp3 phenotype, enhanced angiogenesis, promoted M2 macrophage polarization, and accelerated nerve regeneration, myelination, and functional recovery, highlighting the potential of scaffold-mediated immune modulation in peripheral nerve repair [175].

Beyond scaffold-based methods, cell-free therapies using extracellular vesicles, particularly exosomes (30–150 nm), are emerging as promising tools for neural repair. Nochlabadi et al. highlighted that these vesicles, secreted by various cells, deliver bioactive molecules that promote angiogenesis, reduce inflammation, and support cell proliferation and differentiation. Their small size, biocompatibility, low immunogenicity, and ability to cross the blood–brain barrier make them attractive for nerve regeneration, with ongoing research focusing on enhancing targeting and therapeutic delivery [176].

### 4.6. Liver Tissue Engineering

**Figure 4:**
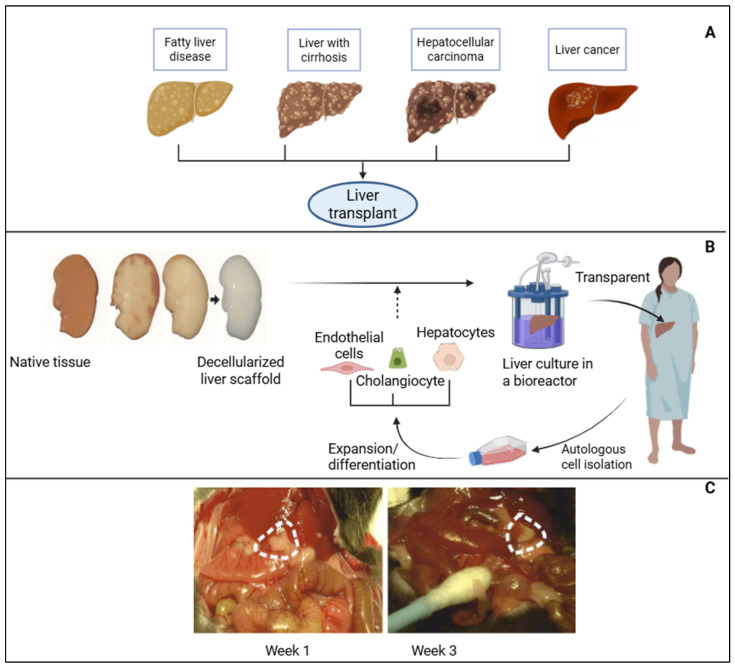
(**A**) Progression of liver diseases leading to the need for transplantation. Conditions such as fatty liver disease, cirrhosis, hepatocellular carcinoma, and advanced liver cancer may ultimately require liver transplant as a treatment option (Created with BioRender.com). (**B**) Schematic representation of bioengineered liver development from decellularized scaffolds. Ongoing decellularization of an individual rat liver lobe is achieved through continuous detergent perfusion, producing an acellular scaffold. This scaffold is recellularized with autologous cells, including hepatocytes, endothelial cells, and cholangiocytes, which are expanded and differentiated in vitro. The seeded scaffold is then matured in a bioreactor to generate a bioengineered liver for potential transplantation into the patient. Panels A and B were designed using BioRender (Created with BioRender.com). (**C**) Macroscopic view of interspecies biocompatibility assessment of human liver cubic scaffolds implanted into the omentum of immunocompetent mice (*n* = 6). Images at weeks 1 and 3 post-implantation show that the scaffold has integrated well with surrounding host abdominal tissues, including the liver, intestine, and pancreas, with no signs of acute rejection or inflammation. (**C**) reprinted from Ref. [177].

Liver TE (LTE) has emerged as a promising approach to address the shortage of donor organs and the limitations of conventional liver transplantation, particularly for patients with advanced or terminal liver diseases such as chronic hepatitis C infection, non-alcoholic fatty liver disease, alcoholic fatty liver disease, decompensated cirrhosis, hepatocellular carcinoma, biliary atresia, and Wilson’s disease (Figure 4A). These conditions, often culminating in cirrhosis and portal hypertension, progressively destroy hepatic structure and function, leaving transplantation as the only curative option in many cases [60]. A key strategy involves the use of decellularized liver extracellular matrix (dECM), which preserves the liver’s native microarchitecture, vascular network, and biochemical composition. Perfusion-based decellularization and recellularization techniques have enabled the engineering of whole-liver grafts with preserved vascular networks, enhancing their transplantation potential. For example, dECM scaffolds derived from human liver tissue have been used as bioinks for 3D bioprinting, creating a physiologically relevant environment for stem cell differentiation and hepatic phenotype induction (Figure 4B) [61].

Overall, LTE strategies integrate functional hepatic cells with bioactive scaffolds and signaling molecules to restore native liver architecture and recover metabolic and synthetic functions. The primary objectives include sustaining long-term hepatocyte viability, maintaining urea and albumin metabolism, and promoting vascularization for efficient oxygen and nutrient delivery [60,178]. Demonstrating interspecies biocompatibility, Mazza et al. implanted human liver cubic scaffolds into the omentum of immunocompetent mice and observed early post-implantation engraftment within host abdominal structures, without signs of acute rejection or inflammation [177].

#### Scaffold Materials and Bioactive Factors in LTE

Scaffolds fabricated from both synthetic and natural polymers have been extensively investigated to mimic the properties of the hepatic ECM while offering tunable degradation rates and mechanical characteristics. For instance, gelatin–alginate composite hydrogels have been shown to support HepG2 cell viability and maintain urea and albumin secretion, with scaffold microarchitecture significantly influencing hepatic function [60]. Similarly, peptide-based hydrogels fabricated via 3D bioprinting recreated hepatic lobule-like spatial arrangements and channel-like pathways for nutrient perfusion, enhancing the metabolic activity of MSCs differentiated into hepatocyte-like cells [178]. Poly (ethylene glycol)-fibrinogen (PEG-Fib) hydrogels supported hepatocyte-like cells with improved hepatic gene expression and metabolic function compared to 2D cultures [178]. Electrospun poly(L-lactide-co-ε-caprolactone)/gelatin (PLCL/Gel) scaffolds promoted cell adhesion and liver-specific function in co-culture with endothelial and hepatocyte cells [62]. Porous scaffolds and microcarriers made from alginate, chitosan, silk fibroin, and PCL have also been explored, sometimes functionalized with RGD peptides or heparin to improve cell attachment and bioactivity. In some cases, these structures were coated with liver-derived ECM or supplemented with growth factors to better replicate the native hepatic microenvironment [62,63]. Bioprinting technologies have played a significant role in creating complex liver-mimetic architectures. For example, GelMA-based bioinks containing hepatocytes and human umbilical vein endothelial cells (HUVECs) have been used to fabricate 3D liver constructs, demonstrating enhanced albumin secretion, CYP450 activity, and bile canaliculi formation over time. The inclusion of endothelial cells improved vascular-like channel formation and reduced hypoxia in larger constructs [62,63].

Combining bioprinting technologies with bioactive scaffold materials, either natural or synthetic, growth factors, and suitable hepatic cell sources can provide an effective platform for functional liver regeneration. However, challenges such as achieving long-term hepatic functionality, complete vascular integration, and protocol standardization for clinical translation remain to be addressed.

### 4.7. Cardiac Tissue Engineering

Cardiac TE has emerged as a promising approach to address the limited regenerative capacity of the adult heart. Cell-based strategies, particularly those utilizing human pluripotent stem cells (hPSCs), have gained increasing attention due to their ability to differentiate into cardiomyocytes (CMs) and other cardiac-relevant cell types. However, several challenges remain in achieving functional and clinically relevant cardiac constructs. A wide range of cell sources have been explored for cardiac regeneration, including MSCs, cardiac progenitor cells, and more recently, hPSC-derived cardiomyocytes (hPSC-CMs). Among these, iPSCs are especially appealing due to their self-renewal capacity and patient-specific compatibility. Advances in differentiation protocols now enable the generation of distinct subtypes of cardiomyocytes, atrial, ventricular, and pacemaker cells, with high purity through modulation of the Wnt/β-catenin pathway [64]. Despite this progress, hPSC-CMs remain phenotypically immature, resembling fetal rather than adult cardiomyocytes in terms of structure, contractile force, metabolism, and electrophysiological properties [64,65]. Immaturity limits their utility in both in vivo therapies and in vitro disease models.

To overcome immaturity, a variety of bioinspired strategies have been developed. Functional maturation has been improved through the application of electrical and mechanical stimulation, co-culture with non-myocyte cells such as fibroblasts and endothelial cells, and exposure to ECM-like environments. Cardiac TE benefits from angiogenic and cardioprotective signals provided by VEGF and hepatocyte growth factor (HGF), which together enhance vascular integration and maintain myocardial viability post-injury [179]. For instance, dynamic culture systems and engineered scaffolds have been used to replicate in vivo mechanical loading, which promotes sarcomere alignment and electrophysiological development [64,65]. Conductive biomaterials have been introduced to facilitate synchronized electrical signaling. Incorporation of gold nanorods (GNRs), carbon-based nanomaterials, and silicon nanowires into hydrogels has enhanced expression of cardiac markers (e.g., Connexin-43, cardiac troponin I), improved calcium handling, and supported synchronous contractions in engineered tissues [66].

#### Scaffold Materials and Bioactive Factors in Cardiac Tissue Engineering

Recent advances in cardiac TE have brought together bioprinting technologies and smart biomaterials to create more functional and physiologically relevant constructs. Among these, 3D bioprinting enables precise spatial control over the distribution of multiple cell types, such as cardiomyocytes, fibroblasts, and endothelial cells, within bioinks that mimic the anisotropic architecture of native myocardium [67]. Techniques like layer-by-layer printing and the incorporation of anisotropic conductive scaffolds have resulted in improved cell alignment, synchronous contractility, and structural fidelity in engineered cardiac tissues. Moreover, the integration of vascular-like channels and supportive stromal cells has significantly enhanced nutrient diffusion, oxygen delivery, and overall viability in thicker constructs, addressing one of the main limitations of avascular tissue models [65,67].

The selection of appropriate materials for scaffold fabrication plays a vital role in the successful functional regeneration of cardiac tissue. To date, two main categories of materials, synthetic polymers and natural or decellularized extracellular matrix (dECM), have been widely explored, each offering distinct advantages and limitations. Among synthetic polymers, materials such as PCL, polyglycerol sebacate (PGS), PU, PLA, and polyglycolic acid (PGA) have been frequently utilized. These polymers are biodegradable, possess favorable mechanical properties, and allow ease of processing. However, they are often hydrophobic and lack cell recognition sites, which compromises cell adhesion and requires further surface modification [68]. Several researchers have attempted to address these limitations. For instance, Tallawi et al. developed PGS/PCL-based composites functionalized with biological coatings such as collagen, laminin, and fibronectin, which significantly enhanced cellular proliferation, tissue alignment, and synchronous contractile behavior [69]. Likewise, Boffito and Ciardelli employed techniques such as electrospinning and bioprinting to fabricate anisotropic scaffold architectures that mimic myocardial tissue. Through chemical and biological surface modifications, including RGD peptide grafting, they improved cellular adhesion and functionality [68].

Decellularized matrices, particularly those derived from human or animal hearts, have gained increasing attention due to their preserved 3D ECM structure and native protein composition. Barbulescu et al. demonstrated that human heart-derived dECM retained vascular architecture, collagen, elastin, and essential ECM components following optimized decellularization processes. These scaffolds supported enhanced stem cell proliferation and differentiation, while also downregulating fibrotic gene expression and upregulating pro-angiogenic markers [70]. Additional studies have shown that cardiac dECM can effectively support cardiomyocytes, fibroblasts, and endothelial cells by providing a bioactive environment, although challenges remain regarding immunogenicity and standardization of decellularization protocols [70].

Roacho-Pérez et al. highlighted that synthetic scaffolds, along with appropriate cell sources and growth factors, represent one of the three essential pillars of cardiac tissue regeneration [71]. They emphasized that effective cardiac scaffolds must not only be biocompatible and biodegradable but also possess electrical conductivity, contractile properties, and the ability to replicate the anisotropic nature of myocardial tissue. In this context, materials such as PCL, gelatin, collagen, fibrin, PPy, graphene, and conductive nanoparticles have been explored, with hybrid formulations like PCL/PPy and collagen/graphene showing potential to enhance electrical conductivity and promote synchronized cardiac tissue formation in electrically responsive environments [71].

Hybrid strategies that combine synthetic polymers with biofunctionalization approaches offer scaffolds integrating the mechanical strength of synthetic materials with the biological activity of natural ECM, supporting effective cardiac regeneration. Recent preclinical and clinical studies (Table 4) highlight these approaches. Several early-phase clinical trials have explored cardiac TE strategies for heart regeneration. The PRESERVATION I (NCT01226563) [180,181,182] and AUGMENT-HF (NCT01311791) [183,184] trials tested injectable hydrogels, IK-5001 and Algisyl-LVR™, respectively, aiming to prevent adverse left ventricular remodeling in heart failure patients, but showed only minor symptomatic improvements without significant structural benefits [64]. Epicardial application of porcine-derived CorMatrix^®^ ECM patches (NCT02887768) [185,186] and intramyocardial injection of the myocardium-specific VentriGel ECM hydrogel (NCT02305602) [187] demonstrated encouraging safety profiles, with VentriGel also showing functional improvement trends in chronic post-MI patients [64]. Cell-based approaches included transplantation of hESC-derived cardiovascular progenitors embedded in fibrin patches for severe ischemic dysfunction (NCT02057900) [188,189], which proved safe but with modest efficacy, and ongoing trials using Wharton’s jelly MSC-seeded ECM patches (NCT04011059) or engineered heart muscle from hiPSC-CMs and stromal cells (NCT04396899) aim to assess both safety and regenerative potential [64]. While most trials reported limited functional gains, they have established feasibility and safety, paving the way for optimized biomaterial-cell combinations and delivery strategies. (Data from ClinicalTrials.gov (https://clinicaltrials.gov/).)

### 4.8. Ophthalmic Applications

TE offers promising therapeutic strategies for several prevalent ocular diseases, including corneal disorders (e.g., keratoconus, limbal stem cell deficiency; ~1 in 500 prevalence), through stromal regeneration using biomaterials, stem cells, collagen cross-linking, and grafting [90,200]; age-related macular degeneration (AMD) (~10% of individuals over 65) via stem cell–derived retinal pigment epithelium (RPE) patches and engineered retinal grafts [200,201]; and diabetic retinopathy (~30% of diabetics) using similar retinal repair approaches [202]. Additional applications include glaucoma (engineered implants to regulate intraocular pressure or regenerate optic nerve fibers) [203], uveitis (localized drug-delivery systems to reduce uveal inflammation) [204], and optic nerve injury (nerve grafts or scaffolds to promote neuronal regeneration) [205]. Collectively, these strategies underscore TE’s potential to restore vision and improve the quality of life in patients with diverse ocular pathologies [90,206].

A variety of cells have been utilized in ophthalmic TE. For conjunctival scaffolds, the main types were conjunctiva mesenchymal stem cells (CJMSCs) and rabbit conjunctival epithelial cells (rCjECs) [72,207,208,209,210]. For corneal scaffolds, studies used human adipose mesenchymal stem cells (hADSCs), limbal stromal stem cells (LSSCs), keratocytes, limbal epithelial progenitor cells (LEPC), limbal mesenchymal stromal cells (LMSC), limbal melanocytes (LM), corneal epithelial cells (CEC, HCECs), corneal fibroblasts (HCFs), corneal endothelial cells (hCEC), human keratocytes (HKs), and rabbit-derived corneal epithelial and stromal cells, as well as hESC-derived corneal epithelial cells (hESC-CEC) [76,77,211]. For lacrimal gland scaffolds, reported cell types included adult rabbit lacrimal gland progenitor cells, lacrimal acinar epithelial cells from Sprague-Dawley rats, porcine lacrimal gland epithelial cells, purified rabbit lacrimal gland acinar cells, and lacrimal gland epithelial cells [86,87,88,89].

Ocular TE utilizes various biomaterials matched to target tissues. Natural materials (e.g., autologous fibrin [212], collagen–GAG composites [76], fibrin hydrogel [213,214]) support epithelialization, mimic native tissue, and enhance nutrient delivery, but may have donor source limitations or reduced durability. Synthetic polymers (e.g., PCL, PLLA, PLGA, PET, PGS [77,79,80] provide mechanical strength, biocompatibility, and guide cell differentiation, though often need surface modification and long-term testing. Biohybrid scaffolds (e.g., collagen/PLCL, GelMA-HA, hSAPN-αHA) combine natural and synthetic advantages, offering ECM mimicry, infection control, and optical improvements, but involve high costs or complex manufacturing [72,208,215,216]. Decellularized tissues (e.g., RHC cornea, lacrimal gland ECM [88,89], optic nerve DON [205]) preserve native architecture and reduce immune rejection yet face donor limitations and functional assessment requirements. A detailed description of these biomaterials, including their sources, properties, and applications, is provided in Table 5.

Two notable clinical studies have explored the application of scaffolds in ocular TE. The first trial (EudraCT no. 2006-006585-42) investigated the use of carbodiimide-crosslinked recombinant human collagen (RHC) implants as corneal scaffolds, aimed at addressing the global shortage of donor corneas [77]. Over a follow-up period of four years, these bioengineered corneas demonstrated stable integration without signs of rejection. Remarkably, recipients did not require the prolonged immunosuppressive therapy typically necessary after donor corneal transplantation. No infiltration of inflammatory dendritic cells was observed in the implant area, whereas patients who received donor corneas, despite immunosuppression, showed central corneal dendritic cell migration during rejection episodes [77]. The implanted RHC corneas also supported gradual regeneration, with nerve and stromal cell repopulation leading to microstructural characteristics similar to healthy corneas. On average, patients achieved a corrected visual acuity of 20/54 at four years, with an improvement of over five Snellen lines. Researchers suggested that using materials with greater mechanical stability could further enhance vision outcomes [77].

The second trial (EudraCT no. 2010-024290-40) involved a nanostructured fibrin–agarose corneal substitute seeded with allogeneic cells to replicate the mechanical, optical, and biological properties of the human anterior cornea [211]. This multi-center, controlled, randomized, open-label study, conducted across ten hospitals in Spain, covers both phase I and II stages. It focuses on adults with severe trophic corneal ulcers unresponsive to conventional therapies or complicated by previous ulcers. The main goal is to evaluate the safety and feasibility of the bioengineered graft, while also collecting evidence of its therapeutic benefit [211]. Safety monitoring includes tracking adverse events, implant stability, infection, and neovascularization. Follow-up assessments are scheduled over 24 months, with 27 post-implant visits at progressively longer intervals [211]. Collectively, these trials illustrate the promise of scaffold-based TE in ocular regeneration. By integrating advances in biomaterials and stem cell science, such approaches could transform ophthalmic care and provide new therapeutic options for complex corneal diseases [208].

## 5. Discussion

Tissue engineering has progressed remarkably over the past two decades, evolving from simple 2D scaffolds to sophisticated 3D and even 4D constructs capable of dynamic interaction with biological and physical cues [121]. A broad range of biomaterials—including natural polymers (e.g., collagen, chitosan), synthetic polymers (e.g., PLGA, PCL, PU), and their hybrids—have been explored to balance biocompatibility, mechanical strength, and controlled degradation. Smart scaffolds, responsive to stimuli such as pH, temperature, or mechanical loading, represent one of the most significant advances, while nanotechnology has enabled reinforcement with bioactive particles and incorporation of conductive or piezoelectric elements [19,223]. Among these advances, 4D bioprinting has emerged as a transformative approach by incorporating time as a functional dimension, enabling engineered constructs to undergo controlled structural or functional changes in response to environmental stimuli. Unlike conventional 3D-printed scaffolds, 4D systems utilize stimuli-responsive materials such as shape-memory polymers and smart hydrogels that can adapt to temperature, pH, hydration, or mechanical forces after implantation. This dynamic adaptability allows constructs to improve tissue integration, promote guided cell alignment, and enhance vascularization in vivo. Recent studies have demonstrated applications of 4D bioprinting in musculoskeletal, cardiac, and soft tissue regeneration, where controlled deformation and remodeling better mimic physiological processes. However, challenges remain in achieving predictable transformation kinetics, long-term mechanical stability, and regulatory standardization [224,225,226]. Together, these platforms support cell survival, differentiation, vascularization, and bioelectric signaling. The integration of growth factors, stem cells, and computational tools such as AI is further accelerating scaffold design and translation [19,223]. Despite these innovations, persistent barriers, including inadequate vascularization, immune reactions, and variability in stem cell behavior still limit consistent clinical translation [224].

### 5.1. Current Challenges and Limitations in Tissue Engineering

Despite substantial advances across diverse tissue applications, several recurring challenges continue to limit the consistent clinical translation of tissue engineering strategies. One of the most critical barriers is inadequate vascularization in large or complex constructions, which restricts oxygen and nutrient diffusion and compromises long-term graft survival. Immune reactions and foreign body responses further complicate host integration, particularly when synthetic or hybrid biomaterials are used. Mechanical mismatch between engineered scaffolds and native tissues may lead to structural instability or implant failure, especially in load-bearing environments such as bone and cartilage regeneration. In addition, variability in stem cell sources, differentiation potential, and degradation kinetics contribute to inconsistent therapeutic outcomes and reduced reproducibility across studies. Scalability, cost, and regulatory approval remain major translational obstacles. The absence of standardized manufacturing protocols limited large-scale production capacity, and complex regulatory pathways significantly slowed the transition from laboratory research to clinical implementation. Addressing these interconnected limitations will be essential to ensure reliable, safe, and economically viable regenerative therapies. These key challenges and limitations are summarized in Table 6.

Across different tissue types, common translational bottlenecks emerge despite biological differences. Vascularization remains the dominant limitation in metabolically active tissues such as liver and cardiac constructs, whereas mechanical mismatch is more critical in load-bearing tissues such as bone and cartilage. Immune modulation and long-term functional integration represent universal challenges. These cross-tissue parallels suggest that future progress in tissue engineering will depend less on tissue-specific solutions alone and more on platform technologies that address shared biological and regulatory constraints.

Different tissues highlight these challenges in unique ways. In skin TE, scaffold systems based on natural polymers (collagen, gelatin, chitosan) or synthetic ones (PCL, PLGA, PU) have improved vascularization, mechanical strength, and stability, especially in hybrid forms [40,102,105]. Cellular strategies using keratinocytes, fibroblasts, MSCs, and ADSCs, combined with growth factors such as EGF, VEGF, bFGF, and TGF-β1, further enhance angiogenesis and dermal remodeling [43,92,93,94,119,120]. Yet poor host integration, limited sensory recovery, and insufficient vascularization in extensive wounds remain obstacles. Pre-vascularized scaffolds, immunomodulatory hydrogels, and patient-specific iPSCs offer promising solutions [101,119]. Looking forward, automated bioreactors, robotic systems, and 3D/4D bioprinting with optimized bioinks may standardize production, while smart biomaterials and AI-assisted design could generate patient-specific constructs with pigmentation, vascularization, and appendages, reducing reliance on autografts and expanding applications to congenital naevi, vitiligo, and epidermolysis bullosa [225,226].

For bone regeneration, significant advances have been made in repairing critical-sized bone defects by combining progenitor cells, bioactive molecules, and scaffolds [227]. SSCs and GFs such as BMP-2, VEGF, PDGF, and IGF are central to osteogenesis and angiogenesis, while co-delivery strategies help recreate the complex bone-healing environment. However, clinical application of BMP-2 has been associated with dose-dependent adverse effects, including ectopic bone formation, inflammatory complications, and soft tissue swelling, which have limited its widespread and standardized clinical use [137,139]. Scaffold design now integrates biocompatibility, tunable mechanics, and bioactivity through hybrid polymers, nanoparticles (e.g., nano-hydroxyapatite, nanoclay) [15,47,151]. Incorporating conductive and piezoelectric materials enables electrical stimulation, which activates pathways such as BMP and Wnt/β-catenin to further promote osteogenesis and angiogenesis [152]. Despite these advances, translation remains limited. Scaffolds often fail mechanically, trigger immune responses, or lack vascular integration, while variability in stem cell behavior and degradation rates adds further complexity. Pre-vascularization strategies like flap prefabrication and axial vascularization improve outcomes but add surgical complexity and donor-site morbidity [227]. Moving forward, multifunctional scaffolds that combine structural strength with controlled delivery of osteogenic and angiogenic cues, immune modulation, and electrical stimulation will be key to developing clinically reliable bone tissue engineering solutions for orthopedic and dental regeneration.

Cartilage engineering highlights the difficulty of replicating the layered architecture and biomechanical demands of native tissue [228]. Natural biomaterials provide biocompatibility but insufficient strength, while synthetic ones offer mechanical reproducibility but limited biological cues. Composite scaffolds aim to bridge these gaps, but host integration, immune reactions, and stability remain challenges. Advances in cell sourcing (chondrocytes, MSCs, iPSCs) and control of environmental factors such as growth factors, oxygen, and mechanical loading have improved outcomes. Nevertheless, TGF-β–mediated chondrogenesis carries the risk of hypertrophic differentiation and endochondral ossification, which may compromise long-term cartilage stability and functional durability [52,229]. However, achieving long-term, structurally durable cartilage regeneration for disorders like osteoarthritis remains elusive [229].

Regeneration of the periodontium is particularly complex, as both hard and soft tissues must be rebuilt in a microbe-rich, mechanically stressed oral environment [230]. Guided tissue and bone regeneration membranes show potential but often degrade prematurely, fail mechanically, or become infected [231]. Hybrid strategies combining collagen or nanofiber scaffolds with growth factors, stem cells, or platelet-rich plasma have improved outcomes [163,166], yet predictable and durable restoration of tooth-supporting structures is still elusive. Beyond the periodontium, whole-tooth regeneration represents an even greater challenge. The “processed tooth” concept requires precise control of key parameters, including identification of master regulatory genes, architectural fidelity (shape, size, color), development of culture systems capable of producing vascularized and innervated constructs, in vivo enamel regeneration, and long-term functional stability against acid attack and oral disease [232]. While advances in biomaterials, stem cell biology, and biofabrication have brought this vision closer to reality, fully functional and clinically reliable dental regeneration remains an ambitious but promising frontier [171].

Cardiac TE has made encouraging progress, with constructs showing improved organization, gene expression, and electrophysiological activity. However, poor survival and engraftment of transplanted cells, immune rejection, and arrhythmogenic risks limit translation [65]. Moreover, variability among iPSC lines and differentiation protocols hampers reproducibility, and hPSC-derived cardiomyocytes remain immature compared to native tissue. Platforms such as “heart-on-a-chip” systems and large-animal models provide valuable insights, but scalability, cost, and standardization remain obstacles. Future advances will depend on integrating patient-specific iPSCs with smart biomaterials, developmental biology insights, and scalable biofabrication [64].

Neural TE presents an even more delicate challenge. The brain is incredibly soft and has a very strict immune environment. Implants often trigger microglial activation and scar formation, blocking communication and impairing function. Hydrogels offer promise by mimicking brain mechanics and delivering regenerative signals, but conductivity is essential for neural communication. To address this, scaffolds are increasingly combined with conductive materials such as graphene and PPy, which provide conductivity, hydrophilicity, and tunability [57]. Natural polymers (collagen, gelatin, chitosan) and synthetic ones (PLGA, PCL) are widely used, often fabricated via electrospinning or 3D bioprinting to guide axonal regrowth. Immunomodulatory scaffolds that promote M2 macrophage polarization or incorporate regulatory T cells with growth factor–loaded hydrogels further improve outcomes [174]. Cell-free approaches such as exosomes are also emerging, capable of reducing inflammation, delivering regenerative signals, and crossing the blood–brain barrier [176]. While these advances highlight a multifaceted strategy, most remain at the preclinical stage, and ensuring long-term safety, integration, and reproducibility will be critical for clinical translation.

Significant progress has been achieved in liver regeneration by combining functional hepatocytes with biomimetic scaffolds such as decellularized liver ECM, gelatin–alginate hydrogels, PEG–fibrinogen composites, and peptide-based bioinks [60,61]. These materials support hepatocyte viability, metabolic activity, and cytochrome P450 function [62]. Bioprinting technologies have further enabled the recreation of lobule-like structures, improving perfusion and stem cell–derived hepatocyte maturation. Functionalization with peptides, heparin, and growth factors such as VEGF and HGF enhances vascularization and metabolic activity [62]. Yet sustaining long-term hepatic function and generating stable, perfusable vascular networks remain key obstacles, alongside the need to standardize bioprinting protocols for regulatory approval. Future directions include integrating pre-vascularized scaffolds with perfusion bioreactors, adopting multicellular co-cultures, and validating these strategies in large-animal models to accelerate the translation of engineered liver grafts [61]. In addition, large-scale manufacturing of dECM-based constructs and the prevention of thrombosis within engineered vascular networks represent major translational barriers, particularly for clinically relevant graft sizes.

Research in ocular regeneration is also accelerating, with scaffold-based strategies showing potential to restore vision in diverse eye conditions [85,233]. Scaffold-based strategies are attractive for their ability to restore vision and improve patient outcomes, though long-term performance and clinical validation remain uncertain [234]. Current studies are assessing scaffold durability and therapeutic effects, while acknowledging that not all injuries or diseases may be suitable targets. The choice of scaffold design and composition is still debated, and robust clinical trials are required to establish efficacy and safety [211]. Although different scaffold types—natural, synthetic, biohybrid, and decellularized—provide unique strengths in mimicking the ECM and supporting regeneration, challenges such as immune reactions, poor integration, high manufacturing costs, and inconsistent degradation limit translation. Overcoming these barriers will be essential for scaffold-based ocular regeneration to advance into reliable therapies for patients with vision disorders [211].

### 5.2. Future Direction

Looking ahead, the future of tissue engineering will depend on overcoming key barriers that currently limit translation into reliable therapies. Standardization of scaffold design, cell sourcing, and preclinical testing is essential for reproducibility and regulatory approval. At the same time, the growing focus on personalized medicine emphasizes tailoring therapies through autologous stem cells, patient-specific iPSCs, and 3D-bioprinted constructs adapted to genetic and immunological profiles. Multifunctional scaffolds capable of providing structural support while delivering drugs, growth factors, or diagnostic nanoparticles may evolve into integrated therapeutic platforms. Advances in nanotechnology, bioelectronics, and computational modeling will further accelerate this progress by improving bioactivity, enabling mechanical or electrical stimulation, and predicting scaffold performance in vivo. In particular, AI and machine learning are emerging as powerful tools in tissue engineering by enabling predictive modeling of scaffold performance, biomaterial selection, and cellular responses. AI-driven algorithms can analyze large experimental datasets to optimize mechanical properties, degradation kinetics, and growth factor delivery strategies, thereby reducing trial-and-error experimentation. Deep learning approaches are also being applied to automate image-based assessment of tissue regeneration and vascularization, improving reproducibility and quantitative evaluation. Furthermore, generative design models may support the development of patient-specific scaffolds tailored to anatomical and biological parameters. Despite these promising applications, challenges related to data standardization, model interpretability, and regulatory validation must be addressed before AI-integrated tissue engineering platforms can achieve widespread clinical adoption. Ultimately, successful translation will require close collaboration among researchers, clinicians, industry, and regulators to bridge the gap between discovery and clinical practice. Together, these innovations could establish tissue engineering as a cornerstone of regenerative medicine, addressing some of the most complex and debilitating diseases of our time.

## 6. Conclusions

TE has evolved into a highly interdisciplinary field capable of addressing complex clinical challenges across multiple organ systems, including skin, bone, cartilage, dental, cardiac, neural, hepatic, and ocular tissues. As highlighted throughout this review, the integration of advanced biomaterials, stem cell-based strategies, nanotechnology, and biofabrication techniques such as 3D and 4D bioprinting has substantially improved scaffold functionality and biological performance. Hybrid scaffolds combining natural and synthetic polymers have emerged as particularly promising platforms, offering a balance between mechanical stability and bioactivity tailored to tissue-specific requirements.

However, despite encouraging preclinical and early clinical outcomes, several critical barriers continue to limit widespread clinical translation. Recurrent challenges such as inadequate vascularization, immune-mediated responses, mechanical mismatch, stem cell variability, scalability, and regulatory complexity remain consistent across different tissue applications. This review underscores that overcoming these limitations will require not only material innovation but also improved standardization, predictive design strategies, and integration of computational tools, including artificial intelligence, to enhance reproducibility and patient-specific optimization.

Looking forward, the future of TE will likely depend on the development of multifunctional, smart, and immunomodulatory scaffolds capable of dynamic adaptation to the host environment, combined with scalable manufacturing systems and regulatory alignment. A shift from proof-of-concept models toward standardized, clinically validated platforms will be essential. Through coordinated collaboration among researchers, clinicians, industry, and regulatory bodies, TE has the potential to transition from experimental promise to reliable therapeutic reality, fundamentally reshaping regenerative medicine.

## Figures and Tables

**Table 1:** Overview of biomaterials, growth factors, and cell sources used in tissue engineering.

Tissue Type	Scaffold Types/Materials	Growth Factors	Cell Sources	Key Study Findings	Main Limitation	Ref.
Skin TE	Collagen sponges, silk fibroin scaffolds, gelatin/chitosan cryogels, PLGA nanofibers, PCL, hydrogels (alginate, HA, PEG), decellularized dermal matrices, composite scaffolds (PCL–collagen, silk fibroin–PU	VEGF, bFGF, EGF, TGF-β1	Keratinocytes, fibroblasts, adipose cells, vascular endothelial cells, Schwann cells	bFGF-loaded collagen sponges and silk fibroin scaffolds enhanced dermal regeneration. TGF-β1 hydrogels reduced inflammation. MSC-based scaffolds improved epithelialization and vascularization.	Limited vascular integration in large wounds; variability in stem cell behavior.	[18,40,41,42,43,44,45]
Bone TE	PLGA, PCL, PLA, collagen, chitosan, alginate, β-TCP, nHA composites, gradient pore scaffolds	BMPs, VEGF TGF-β, PDGFs, IGFs	SSCs, MSCs	BMP-2 PLGA/GelMA scaffolds enhanced bone mineralization. VEGF + BMP-2 promoted angiogenesis and osteogenesis. nHA, TiO_2_, and Ag nanoparticles improved osteoconductivity.	Mechanical mismatch in load-bearing defects; risk of ectopic ossification with BMP-2.	[16,46,47,48,49,50]
Cartilage TE	Chitosan–collagen, alginate, HA hydrogels, GelMA, bioinks, decellularized cartilage matrix	TGF-β BMP-2 IGF-1	BMSCs, chondrocytes	Bioprinted GelMA + BMSC scaffolds repaired full-thickness defects. TGF-β1 improved matrix deposition. BMP-7 enhanced cartilage-specific gene expression.	Poor long-term integration and mechanical durability.	[51,52,53]
Dental & Periodontal Applications	Collagen, chitosan, hyaluronic acid, fibrin, PLGA, GelMA Decm	PDGF, TGF-β, FGF, BMPs, VEGF	DPSCs, hPDLSCs, PDL stem cells	PRF enhanced root development. PDGF-BB PLGA scaffolds improved cementum-like tissue. dECM scaffolds promoted adhesion, proliferation, and differentiation.	Complex multi-tissue interface regeneration remains unpredictable.	[54,55,56]
Nerve TE	Collagen, gelatin, chitosan conduits, PLGA, PCL, graphene, PPy	NGF, BDNF, NT3, GDN, FGF, IGF, CNTF, GH	iPSC, MSCs, Schwann cells, NSCs	Electrospun PLGA + Schwann cells improved axonal regrowth. Conductive scaffolds promoted neurite outgrowth and functional recovery.	Limited functional recovery across long-gap nerve defects.	[57,58,59]
Liver TE	dECM, gelatin–alginate, PEG-Fib PLCL/gelatin, alginate, chitosan silk fibroin, PCL	VEGF, HGF EGF, Bfgf, TGF-β, TNF-α, 6 (IL-6)	MSCs, iPSCs, ESCs, HPCs, fetal stem cells	dECM bioinks promoted hepatic differentiation. GelMA-HUVEC-hepatocyte constructs improved albumin secretion and vascular channel formation.	Difficulty maintaining long-term hepatocyte function and stable perfusable vasculature.	[60,61,62,63]
Cardiac TE	PCL, PGS, PU, PLA, PGA collagen, gelatin, fibrin PPy, graphene, dECM, PU-GNT/NW composite scaffolds, AuNPs–Cs thermosensitive hydrogels, fullerenol/alginate hydrogel, PCL/PEG/MWCNTs with FG-coated nanocomposite scaffolds	VEGF FGF IGF (implied via ECM mimicking and vascularization support)	hPSC-CMs, iPSCs, fibroblasts, endothelial cells	PGS/PCL composites with collagen/laminin improved proliferation and alignment. -Cardiac dECM supported differentiation and angiogenesis. -PU-GNT/NW composite scaffolds—H9C2 rat cardiomyocytes → more native morphology, increased proliferation, and higher expression of cardiac differentiation genes. -AuNPs–Cs thermosensitive hydrogels—MSCs → supported. -PCL/PEG/MWCNTs with FG-coated nanocomposite scaffolds—mouse myoblasts → increased electrical conductivity, wettability, distribution, and development of myoblasts. -GelMA-AuNWs hybrid hydrogels—neonatal rat cardiomyocytes → enhanced biological activity, synchronous contractions, and faster spontaneous beating. PGS/PCL with collagen/laminin improved proliferation and alignment; cardiac dECM promoted differentiation and angiogenesis; conductive composites enhanced synchronized contractions.	Limited electrical integration and risk of arrhythmia, poor long-term cell survival and engraftment, and lack of large-scale vascularization restrict clinical translation.	[64,65,66,67,68,69,70,71,72,73,74,75]
Cornea	Microgrooved collagen films, collagen–GAG composites, RHC implants (carbodiimide crosslinked), nanostructured fibrin–agarose	EGF, FGF, VEGF, IGF, TGF	hADSCs, LSSCs, LEPC, LMSC, LM, CEC, HCECs, HCFs, HKs, hESC-CEC	Optical clarity supports stromal and nerve regeneration; RHC clinical trial (EudraCT 2006-006585-42) showed stable 4-year integration, no rejection, improved vision; fibrin–agarose trial (EudraCT 2010-024290-40) demonstrated safety and feasibility in refractory ulcers	Long-term transparency, innervation, and immune compatibility remain challenging; standardized clinical-grade cell sourcing and manufacturing are still limited.	[76,77,78]
Retina	PCL, PLLA, PLGA, PET, PGS, POC, RWSF/PCL, gelatin/chitosan, HAMP/PCL	BDNF, CNTF, VEGF, PEDF	Photoreceptor precursors, MSCs	Biocompatible scaffolds guide photoreceptor differentiation; ECM-like structure with optimal porosity; needs long-term evaluation	Functional integration (synaptic connectivity), correct laminar organization, and long-term survival of transplanted cells are difficult; immune responses and delivery/implantation constraints persist.	[79,80,81]
Optic Nerve	Netrin-1 gradient + electrospun scaffold, PCL, PBG, PEDOT-coated PCL	NGF, brain-derived nerve growth factor, neurotrophin-3, and neurotrophin-4/5	RGCs, neuronal stem cells	Guides axon growth and elongation; enhances survival; has potential in glaucoma therapy; has undergone limited human trials	Long-distance axon regeneration and reconnection to central targets remain limited; achieving meaningful visual function recovery and translation beyond small animal models is challenging.	[82,83,84]
Lacrimal Gland	Polyester membranes (PES), ECM from NZW LG, SIS, DC-LG	EGF, FGFs, HGF, and Keratinocyte Growth Factor (KGF)	Lacrimal acinar epithelial cells (rabbit, rat, porcine)	Strong mechanical properties, maintains epithelial polarity; limited functional assessment	Restoring sustained tear secretion and functional innervation/duct architecture is difficult; most studies lack long-term functional assessment and clinical validation.	[85,86,87,88,89]
Lens	HA + nondegradable gel	FGF and TGF-β	Lens epithelial cells	Improved lens transparency; requires long-term stability studies	Maintaining long-term optical clarity and refractive function is challenging; risks include fibrosis/opacification and limited in vivo validation/standardization.	[90]

**Table 4:** Preclinical and clinical studies in cardiac TE.

Year	Study Type	Model/ Condition	TE Strategy	Material/ Approach	Cell Source	Key Outcomes	Ref.
2015	In vivo preclinical	Rat MI (IR 60 min)	Engineered heart tissue	CM and collagen type I macroscale ring EHT	hESC-CM	No significant changes in LVEDV and LVESV at 4 weeks; progressive improvement in LVEF at 4 weeks, assessed by echocardiography and MRI	[190]
2015	In vivo preclinical	Mouse MI	Cardiac patch	3D-printed patch composed of HA/gelatin-based matrix	Human CPC	Reduction in LVEDV and LVESV at 4 weeks; reduction in infarct fibrosis, assessed by MRI and histology	[191]
2016	In vivo preclinical	Guinea pig (cryo-injury)	Engineered heart tissue	Fibrin EHT	hiPSC-CM,-EC	Improved LV function at day 28; EHT vascularization and electrical coupling with host heart tissue; assessed by echocardiography	[192]
2016	Preclinical	Pig (MI, IR 60 min)	Cardiac patch	Cardiac muscle patch	hiPSC-CM, -EC, and SMCs	LVEF and LVEDV improvements at 4 weeks; no spontaneous arrhythmias detected 2 weeks post-acute MI; assessed by MRI and electrocardiograph	[193]
2016	Clinical trial	Acute myocardial infarction; congestive heart failure; ST-elevation myocardial infarction	Injectable hydrogel therapy	IK-5001 (sodium alginate and calcium gluconate hydrogel)	N/I	Phase 1 trial completed; evaluated prevention of ventricular remodeling and HF progression	[180]
2017	Preclinical	Rat	Cardiac patch	Fibrin patch with nylon frame	hiPSC-CM	Patches did not achieve electrical coupling with recipient hearts, showed vascularization by host vessels, and no immune rejection was observed	[194]
2017	Preclinical	Rat	Nanofibers	Poly (D,L-lactic-co-glycolic acid) polymer nanofibers	hiPSC-CM	Four-week follow-up showed increased LVEF, LVFS, and LVESD; no immune rejection was observed, assessed via echocardiography and histology	[195]
2018	Clinical trial	Ischemic heart disease	Cardiac patch	Fibrin patch containing hESC-derived CD15^+^Isl-1^+^ progenitor cells	hESC-derived progenitor cells (CD15^+^Isl-1^+^)	Phase 1 study completed; evaluated the safety and feasibility of progenitor cell transplantation using a fibrin patch in severe heart failure patients	[188]
2018	Preclinical	Pig	Cardiac patch	3D fibrin patch loaded with insulin growth factor-encapsulated microspheres	hiPSC-derived CM, EC, and SMC	Improved LVEF at 4 weeks; reduced LV wall stress; decreased infarct size; outcomes assessed via echocardiography and histology	[196]
2019	Preclinical	Rat model of MI	Cardiac patch	Viscoelastic starch patch designed using finite-element simulation	Acellular	Decreased LVIDD and LVIDS; improved LVEF and LVFS at 4 weeks; reduced infarct size; reduced myocardial hypertrophy; assessed by echocardiography, histology, and immunofluorescence microscopy	[197]
2019	Preclinical	Guinea pig cryo-injury model	Engineered heart tissue implantation	Fibrin-engineered heart tissue	hiPSC-CM, hiPSC-EC	No pro-arrhythmogenic effects at day 28; assessed by echocardiography	[198]
2022	Preclinical	Rat (neonatal cardiomyocytes)	Cardiac patch	PEG-poly (DL- lactide) (PELA)	Neonatal rat cardiomyocytes (NRCMs) and endothelial cells	The scaffold demonstrated superior mechanical strength, improved cell viability, and extensive capillary-like network formation, with spontaneous beating rates comparable to those seen in adult or neonatal rats	[85]
2023	Preclinical	Rat	Injectable hydrogel therapy (intramyocardial delivery)	Hydrogel composed of chitosan and HA	ASMCs	Improved left ventricular ejection fraction, enhanced fractional shortening, promoted new vessel growth, and demonstrated high mechanical strength and cell retention. Limitations: Complex fabrication process and possible immune reaction	[199]

**Table 5:** Summary of biomaterials used in ocular TE.

Biomaterial Type	Target Tissue	Material Examples	Advantages	Disadvantages	Ref.
Natural	Conjunctiva	Autologous fibrin	Anti-inflammatory, promotes epithelialization	Limited to the patient’s source	[212]
	Cornea	Microgrooved collagen films, collagen–GAG composites	Optical clarity mimics natural swelling, reduces fibrosis	Needs improved mechanical durability	[76]
	Retina	Cask, Caskin1, RS1, GCH composite, fibrin hydrogel	Supports cell survival, integration into host tissue, and improves oxygen/nutrient delivery	Limited long-term stability in vivo	[213,214]
	Optic Nerve	Netrin-1 gradient + electrospun scaffold	Guides RGC axon growth, potential for glaucoma therapy	Preclinical stage	[82]
Synthetic	Lacrimal Gland	PES	Strong mechanical & chemical properties	Non-biodegradable	[85,87]
	Retina	PCL, PLLA, PLGA, PET, PGS, POC	Biocompatible, directs stem cell differentiation to photoreceptors	Requires surface modifications for better integration	[78,79,80]
	Optic Nerve	PCL, PBG, PPy-G, PEDOT-coated PCL	Enhances axon survival and elongation, guides nerve bundles	Limited human trials	[83,84]
Biohybrid	Conjunctiva	PLA + CNF/SP-LF, SF/rGO, collagen/PLCL	Antibacterial, biocompatible, stratified epithelium formation	High production cost	[18,72,207,208]
	Cornea	PeCL + Collagen I, SF + GDNF, GelMA-HA, PVA-COL, VH, sHAPN, oHA	ECM mimicry, anti-inflammatory, infection control	Complex manufacturing	[210,215,216,217,218]
	Lens	HA + nondegradable gel	Improved lens transparency	Requires long-term stability studies	[90]
	Retina	RWSF/PCL/Gelatin, Gelatin/Chitosan, HAMP/PCL	ECM-like structure, optimal porosity, high biocompatibility	Needs long-term evaluation	[81,210,216]
Decellularized Tissue	Cornea	LCs, FD-APCS, SMILE lenticule, AM, RHC, DHC	Non-immunogenic, preserves collagen structure	Limited donor source	[77,219,220]
	Lacrimal Gland	ECM from NZW LG, SIS-Muc, DC-LG	Maintains epithelial polarity pattern	Requires long-term functional assessment	[86,88,89]
	Optic Nerve	DON, porcine DON	Removes axon inhibitory factors, increases axon length and branching	Limited clinical testing	[221,222]

**Table 6:** Major challenges and limitations in tissue engineering.

Challenge	Underlying Cause	Impact on Clinical Translation	Emerging Strategies
Poor vascularization	Limited nutrient and oxygen diffusion in large constructs	Necrosis, graft failure	Pre-vascularized scaffolds, angiogenic factor delivery
Immune response	Foreign body reaction, inflammation	Fibrosis, poor integration	Immunomodulatory biomaterials, controlled degradation
Mechanical mismatch	Inadequate scaffold stiffness or degradation rate	Structural instability, implant failure	Hybrid/composite scaffolds, smart materials
Stem cell variability	Donor heterogeneity, inconsistent differentiation	Unpredictable outcomes	Standardized cell sourcing, iPSC technologies
Scalability and manufacturing	Complex fabrication and lack of standardization	High cost, limited reproducibility	Automated bioreactors, process optimization
Regulatory barriers	Lack of harmonized guidelines and long approval pathways	Delayed clinical translation	GMP protocols, translational regulatory frameworks

## Data Availability

No new data were created or analyzed in this study. Data sharing is not applicable to this article.

## Abbreviations

The following abbreviations are used in this manuscript: Artificial Intelligence (AI); Adipose-Derived Stem Cell (ADSC); Brain-Derived Neurotrophic Factor (BDNF); Bone Morphogenetic Protein (BMP); Bone Marrow-Derived Stem Cell (BMSC); Ciliary Neurotrophic Factor (CNTF); Dental Pulp Stem Cell (DPSC); Extracellular Matrix (ECM); Epidermal Growth Factor (EGF); Embryonic Stem Cell (ESC); Fibroblast Growth Factor (FGF); Basic Fibroblast Growth Factor (bFGF); Glial Cell-Derived Neurotrophic Factor (GDNF); Growth Hormone (GH); Hyaluronic Acid (HA); Hepatic Progenitor Cell (HPC); Human Periodontal Ligament Stem Cell (hPDLSC); Insulin-Like Growth Factor (IGF); Induced Pluripotent Stem Cell (iPSC); Mesenchymal Stem Cell (MSC); Nerve Growth Factor (NGF); Neural Stem Cell (NSC); Neurotrophin-3 (NT3); Poly(ε-caprolactone) (PCL); Platelet-Derived Growth Factor (PDGF); Polyethylene Glycol (PEG); Polyethersulfone (PES); Poly(glycolic acid) (PGA); Poly(glycerol sebacate) (PGS); Poly(lactic acid) (PLA); Poly(lactic-co-glycolic acid) (PLGA); Polyvinylpyrrolidone (PVP); Small Intestinal Submucosa (SIS); Stereolithography (SLA); Selective Laser Sintering (SLS); Tissue Engineering (TE); Transforming Growth Factor Beta (TGF-β); Vascular Endothelial Growth Factor (VEGF).
